# COVID-19 Vaccine Safety in Adolescents Aged 12–17 Years — United States, December 14, 2020–July 16, 2021

**DOI:** 10.15585/mmwr.mm7031e1

**Published:** 2021-08-06

**Authors:** Anne M. Hause, Julianne Gee, James Baggs, Winston E. Abara, Paige Marquez, Deborah Thompson, John R. Su, Charles Licata, Hannah G. Rosenblum, Tanya R. Myers, Tom T. Shimabukuro, David K. Shay

**Affiliations:** ^1^CDC COVID-19 Response Team; ^2^Food and Drug Administration, Silver Spring, Maryland; ^3^Epidemic Intelligence Service, CDC.

As of July 30, 2021, among the three COVID-19 vaccines authorized for use in the United States, only the Pfizer-BioNTech BNT162b2 mRNA COVID-19 vaccine is authorized for adolescents aged 12–17 years. The Food and Drug Administration (FDA) issued an Emergency Use Authorization (EUA) for Pfizer-BioNTech vaccine for use in persons aged ≥16 years on December 11, 2020 (*1*); the EUA was expanded to include adolescents aged 12–15 years on May 10, 2021 (*2*), based on results from a Phase 3 clinical trial (*3*). Beginning in June 2021, cases of myocarditis and myopericarditis (hereafter, myocarditis) after receipt of Pfizer-BioNTech vaccine began to be reported, primarily among young males after receipt of the second dose (*4*,*5*). On June 23, 2021, CDC’s Advisory Committee on Immunization Practices (ACIP) reviewed available data and concluded that the benefits of COVID-19 vaccination to individual persons and the population outweigh the risks for myocarditis and recommended continued use of the vaccine in persons aged ≥12 years (*6*). To further characterize safety of the vaccine, adverse events after receipt of Pfizer-BioNTech vaccine reported to the Vaccine Adverse Event Reporting System (VAERS) and adverse events and health impact assessments reported in v-safe (a smartphone-based safety surveillance system) were reviewed for U.S. adolescents aged 12–17 years during December 14, 2020–July 16, 2021. As of July 16, 2021, approximately 8.9 million U.S. adolescents aged 12–17 years had received Pfizer-BioNTech vaccine.* VAERS received 9,246 reports after Pfizer-BioNTech vaccination in this age group; 90.7% of these were for nonserious adverse events and 9.3% were for serious adverse events, including myocarditis (4.3%). Approximately 129,000 U.S. adolescents aged 12–17 years enrolled in v-safe after Pfizer-BioNTech vaccination; they reported local (63.4%) and systemic (48.9%) reactions with a frequency similar to that reported in preauthorization clinical trials. Systemic reactions were more common after dose 2. CDC and FDA continue to monitor vaccine safety and provide data to ACIP to guide COVID-19 vaccine recommendations.

VAERS is a passive vaccine safety surveillance system comanaged by CDC and FDA that monitors adverse events after vaccination (*7*). VAERS accepts reports from anyone, including health care providers, vaccine manufacturers, and members of the public. Under COVID-19 vaccine EUA requirements, health care providers must report certain adverse events after vaccination to VAERS, including death.^†^ Signs, symptoms, and diagnostic findings in VAERS reports are assigned Medical Dictionary for Regulatory Activities (MedDRA) preferred terms by VAERS staff members.^§^ VAERS reports are classified as serious if any of the following are reported: hospitalization or prolongation of hospitalization, life-threatening illness, permanent disability, congenital anomaly or birth defect, or death.^¶^ Reports of serious adverse events receive follow-up to obtain additional information, including medical records; for reports of death, death certificates and autopsy reports are obtained, if available. CDC physicians reviewed available information for each decedent to form an impression about cause of death.

CDC established v-safe, a voluntary smartphone-based active safety surveillance system, to monitor adverse events after COVID-19 vaccination. Adolescents who receive a COVID-19 vaccine are eligible to enroll in v-safe, through self-enrollment or as a dependent of a parent or guardian, and receive scheduled text reminders about online health surveys.** Health surveys sent in the first week after vaccination include questions about local injection site and systemic reactions and health impacts.^††^ If a report indicated medical attention was sought, VAERS staff members contacted the reporter and encouraged completion of a VAERS report, if indicated.

VAERS and v-safe data were assessed by sex, age group, and race/ethnicity for U.S. adolescents aged 12–17 years who received Pfizer-BioNTech vaccine during December 14, 2020–July 16, 2021. VAERS reports for adolescents aged 12–15 years were excluded if vaccination occurred before EUA age expansion on May 10, 2021. FDA used empirical Bayesian data mining to monitor for disproportional reporting of adverse events by vaccine among VAERS reports for persons aged 12–17 years^§§^ (*8*). SAS software (version 9.4; SAS Institute) was used to conduct all analyses. These surveillance activities were reviewed by CDC and conducted consistent with applicable federal law and CDC policy.^¶¶^

## Review of VAERS Data

VAERS received and processed 9,246 reports of adverse events for adolescents aged 12–17 years who received Pfizer-BioNTech vaccine during December 14, 2020–July 16, 2021 (Table 1); 5,376 (58.1%) were in adolescents aged 12–15 years and 3,870 (41.9%) in persons aged 16–17 years.*** No adverse events were reported disproportionately to VAERS in association with Pfizer-BioNTech vaccination. Common conditions among all reports included dizziness (1,862; 20.1%), syncope (1,228; 13.3%), and headache (1,027; 11.1%). Among the 1,228 reports of syncope, 901 met a standard case definition^†††^; 548 (60.8%) of these events occurred in females, and median age was 15 years. Among those who met the syncope case definition, 147 (16.3%) reported a history of anxiety around needles, and 145 (16.1%) were transported to an emergency department for further evaluation.

**TABLE 1:** Adverse event reports for adolescents aged 12–17 years who received the Pfizer-BioNTech COVID-19 vaccine, by demographic characteristics and reported symptoms (N = 9,246) — Vaccine Adverse Event Reporting System, United States, December 14, 2020–July 16, 2021

Characteristic	Total, % (N = 9,246)	Severity, %*		
		Nonserious (n = 8,383)	Serious, excluding death (n = 849)	Death (n = 14)
**Sex**				
Female	52.9	55.3	29.1	35.7
Male	45.8	43.2	70.7	64.3
Unknown	1.4	1.5	0.2	0
**Age group, yrs**				
12–15	58.1	58.7	53.4	28.6
16–17	41.9	41.3	46.6	71.4
**Ethnicity**				
Hispanic or Latino	10.4	9.6	18.4	7.1
Non-Hispanic or Latino	44.1	43.4	51.2	50.0
Unknown ethnicity	45.5	47.1	30.4	42.9
**Race**				
American Indian or Alaska Native	0.8	0.8	0.5	0
Asian	5.2	5.0	7.3	7.1
Black	3.2	3.0	5.3	7.1
Native Hawaiian or Pacific Islander	0.3	0.2	0.7	0
White	45.1	44.2	53.8	71.4
Multiracial	2.1	2.2	2.0	0
Other	13.1	13.9	5.4	0
Unknown race	30.2	30.8	25.0	14.3

**Abbreviation:** VAERS = Vaccine Adverse Event Reporting System.

* VAERS reports are classified as serious if any of the following are reported: hospitalization or prolongation of hospitalization, life-threatening illness, permanent disability, congenital anomaly or birth defect, or death.

Overall, 8,383 (90.7%) VAERS reports were for nonserious events, and 863 (9.3%) for serious events, including death; 609 (70.6%) reports of serious events were among males, and median age was 15 years. The most commonly reported conditions and diagnostic findings among reports of serious events were chest pain (56.4%), increased troponin levels (41.7%), myocarditis (40.3%), increased c-reactive protein (30.6%), and negative SARS-CoV-2 test results (29.4%) (Table 2); these findings are consistent with a diagnosis of myocarditis. Myocarditis was listed among 4.3% (397) of all VAERS reports.

**TABLE 2:** Most frequent symptoms, signs, diagnostic results, and conditions* reported to the Vaccine Adverse Event Reporting System for adolescents aged 12–17 years after receipt of the Pfizer-BioNTech COVID-19 vaccine (N = 9,246) — United States, December 14, 2020–July 16, 2021

Symptom, sign, diagnostic result, or condition	% Reporting
**Nonserious reports (n = 8,383)**	
Dizziness	21.2
Syncope	14.4
Nausea	10.4
Headache	10.0
Fever	8.3
Loss of consciousness	7.5
Excessive sweating	7.4
Fatigue	7.2
Pallor	7.1
Product administered to patient outside of indicated age range	7.0
Product storage error	6.4
Vomiting	6.4
Difficulty breathing	5.3
Chest pain	4.9
Pain	4.6
**Serious reports, including reports of death**^†^ **(n = 863)**	
Chest pain	56.4
Increased troponin	41.7
Myocarditis	40.3
Increased c-reactive protein	30.6
Negative SARS-CoV-2 test result	29.4
Fever	28.3
Normal echocardiogram	26.9
Abnormal electrocardiogram	25.6
Headache	22.2
Difficulty breathing	21.4
Elevated electrocardiogram ST segment	20.5
Normal chest radiograph	19.7
Intensive care	18.1
Vomiting	17.0
Nausea	16.6

**Abbreviations:** MedDRA = Medical Dictionary for Regulatory Activities; VAERS = Vaccine Adverse Event Reporting System.

* Signs and symptoms in VAERS reports are assigned MedDRA preferred terms by VAERS staff members. Each VAERS report might be assigned more than one MedDRA preferred term, which can include normal diagnostic findings. A MedDRA-coded event does not indicate a medically confirmed diagnosis. https://www.meddra.org/how-to-use/basics/hierarchy

^†^ VAERS reports are classified as serious if any of the following are reported: hospitalization, prolongation of hospitalization, life-threatening illness, permanent disability, congenital anomaly or birth defect, or death.

CDC reviewed 14 reports of death after vaccination. Among the decedents, four were aged 12–15 years and 10 were aged 16–17 years. All death reports were reviewed by CDC physicians; impressions regarding cause of death were pulmonary embolism (two), suicide (two), intracranial hemorrhage (two), heart failure (one), hemophagocytic lymphohistiocytosis and disseminated *Mycobacterium chelonae* infection (one), and unknown or pending further records (six).

## Review of v-safe Data

During December 14, 2020–July 16, 2021, v-safe enrolled 66,350 adolescents aged 16–17 years who received Pfizer-BioNTech vaccine (Table 3). After Pfizer-BioNTech vaccine was authorized for adolescents aged 12–15 years (beginning May 10, 2021), v-safe enrolled 62,709 adolescents in this age group. During the week after receipt of dose 1, local (63.9%) and systemic (48.9%) reactions were commonly reported by adolescents aged 12–15 years; systemic reactions were more common after dose 2 (63.4%) than dose 1 (48.9%). Reporting trends were similar for adolescents aged 16–17 years: systemic reactions were reported among 55.7% after dose 1 and 69.9% after dose 2. For each dose and age group, reactions were reported most frequently the day after vaccination. The most frequently reported reactions for both age groups after either dose were injection site pain, fatigue, headache, and myalgia.

**TABLE 3:** Reactions reported by adolescents aged 12–17 years (N = 129,059) who completed at least one v-safe health check-in survey on days 0–7 after receiving Pfizer BioNTech COVID-19 vaccine — United States, December 14, 2020–July 16, 2021

Event	% of v-safe enrollees reporting reaction or health impact*			
	Age 16–17 yrs, dose (no.)		Age 12–15 yrs, dose (no.)	
	Dose 1 (66,350)	Dose 2 (41,040)	Dose 1 (62,709)	Dose 2 (38,817)
**Any injection site reaction**	62.7	64.4	63.9	62.4
Itching	5.7	6.3	5.8	5.5
Pain	60.2	62.0	61.2	59.9
Redness	3.4	4.9	4.1	5.3
Swelling	7.7	9.9	7.5	8.9
**Any systemic reaction**	55.7	69.9	48.9	63.4
Abdominal pain	4.7	8.5	4.1	7.0
Myalgia	25.4	40.7	21.4	31.4
Chills	8.3	26.2	6.8	21.1
Diarrhea	4.2	4.9	3.1	3.3
Fatigue	34.1	52.3	27.4	44.6
Fever	9.9	31.0	9.3	29.9
Headache	29.8	50.6	25.2	43.7
Joint pain	7.9	18.2	6.3	12.4
Nausea	10.2	19.8	7.5	14.8
Rash	1.2	1.1	1.2	1.2
Vomiting	1.1	2.3	1.0	2.6
**Any health impact**	11.0	28.6	10.6	25.4
Unable to perform normal daily activities	9.0	24.7	9.3	23.1
Unable to work or attend school	3.7	11.6	2.4	6.1
Needed medical care	0.5	0.6	0.5	0.8
Telehealth	0.1	0.2	0.1	0.2
Clinic	0.2	0.2	0.2	0.3
Emergency department visit	0.1	0.2	0.1	0.2
Hospitalization	0.02	0.03	0.02	0.04

* Percentage of enrollees who reported a reaction or health impact at least once during days 0–7 post-vaccination.

During the week after receipt of dose 2, approximately one third of adolescents in both age groups reported fever. Nearly one quarter of adolescents in both age groups reported they were unable to perform normal daily activities the day after dose 2. Fewer than 1% of adolescents aged 12–17 years required medical care in the week after receipt of either dose; 56 adolescents (0.04%) were hospitalized.

## Discussion

The findings summarized in this report are consistent with the safety data observed in preauthorization trials for Pfizer-BioNTech after vaccination among persons aged 12–25 years, with the exception of myocarditis, a serious adverse event detected in postauthorization safety monitoring (*3*). Trial participants who received vaccine (1,131 aged 12–15 years; 537 aged 16–25 years) reported local and systemic reactions that were mostly mild (i.e., did not interfere with activity) or moderate (some interference with activity); no serious adverse events related to vaccination were reported (*3*). Similarly, local and systemic reactions were commonly reported by U.S. adolescents aged 12–17 years who enrolled in v-safe; a minority (<25%) reported they were unable to perform normal daily activities the day after receipt of dose 2. A small number of v-safe participants reported they were hospitalized after vaccination; however, v-safe does not record reason for hospitalization, and it cannot be determined whether hospitalization was related to vaccination.

Among 8.9 million adolescents vaccinated during the study period, VAERS reports were received for approximately one per 1,000 vaccinees, and 90% of these reports were for nonserious conditions. Syncope was among the events most commonly reported to VAERS in this age group and is common among adolescents after any vaccination (*9*). Other conditions associated with vasovagal response to vaccination were also frequently reported. Among the serious reports, myocarditis and other conditions that might be associated with myocarditis were among the most common terms reported; however, these terms did not account for a large proportion of VAERS reports overall. No reports of death to VAERS were determined to be the result of myocarditis. Impressions regarding cause of death did not indicate a pattern suggestive of a causal relationship with vaccination; however, cause of death for some decedents is pending receipt of additional information. ACIP conducted a risk-benefit assessment based in part on the data presented in this report and continues to recommend the Pfizer-BioNTech COVID-19 vaccine for all persons aged ≥12 years (*6*). An updated EUA now includes information on myocarditis after mRNA COVID-19 vaccines.^§§§^

The findings in this report are subject to at least five limitations. First, VAERS is a passive surveillance system and is subject to underreporting and reporting biases (*7*); however, under EUA, health care providers are required to report all serious events following vaccination. Second, medical review of reported deaths following vaccination is dependent on availability of medical records, death certificates, and autopsy reports, which might be unavailable or not available in a timely manner. Third, lack of a statistical safety signal in planned monitoring does not preclude a safety concern. For example, although a statistically significant data mining alert has not been observed for myocarditis following Pfizer-BioNTech vaccination, myocarditis has been identified as an adverse event following mRNA COVID-19 vaccines in multiple surveillance systems (*10*). Fourth, this study was not designed to identify all cases of myocarditis; only reports that listed the MedDRA term “myocarditis” were included. Finally, v-safe is a voluntary self-enrollment program that requires children aged <15 years be enrolled by a parent or guardian and relies on vaccine administrators to promote the program. Therefore, v-safe data might not be generalizable to the overall vaccinated adolescent population.

The initial safety findings of Pfizer-BioNTech vaccine administered to U.S. adolescents aged 12–17 years are similar to those described in the clinical trials, with the exception of myocarditis, a rare serious adverse event associated with receipt of mRNA-based COVID-19 vaccines; follow-up of reported myocarditis cases is ongoing (*6*). CDC and FDA will continue to monitor for adverse events, including myocarditis, after mRNA COVID-19 vaccination and share available data with ACIP to guide risk-benefit assessments for all COVID-19 vaccines.

**Summary**

**What is already known about this topic?**

In preauthorization trials of the Pfizer-BioNTech COVID-19 vaccine, adolescents aged 12–17 years reported local and systemic mild and moderate reactions. Myocarditis has been observed after vaccination with mRNA vaccines in postauthorization monitoring.

**What is added by this report?**

Local and systemic reactions after vaccination with Pfizer-BioNTech vaccine were commonly reported by adolescents aged 12–17 years to U.S. vaccine safety monitoring systems, especially after dose 2. A small proportion of these reactions are consistent with myocarditis.

**What are the implications for public health practice?**

Mild local and systemic reactions are common among adolescents following Pfizer-BioNTech vaccine, and serious adverse events are rare. The Advisory Committee on Immunization Practices conducted a risk-benefit assessment and continues to recommend the Pfizer-BioNTech COVID-19 vaccine for all persons aged ≥12 years.

## Footnotes

*
https://covid.cdc.gov/covid-data-tracker/#datatracker-home

†
https://vaers.hhs.gov/faq.html

§ Each VAERS report might be assigned more than one MedDRA preferred term. A MedDRA-coded event does not indicate a medically confirmed diagnosis. https://www.meddra.org/how-to-use/basics/hierarchy

¶ Based on the Code of Federal Regulations Title 21. https://www.accessdata.fda.gov/scripts/cdrh/cfdocs/cfcfr/cfrsearch.cfm?fr

** Adolescents aged <15 years must be enrolled by a parent or guardian and may not self-enroll. Health check-ins are sent via text messages that link to web-based surveys on days 0–7 after vaccination; then weekly through 6 weeks after vaccination; and then 3, 6, and 12 months after vaccination.

†† Participants in v-safe self-identify the severity of their symptoms, defined as mild (noticeable, but not problematic), moderate (limit normal daily activities), or severe (make daily activities difficult or impossible). Health impacts include whether the vaccine recipient was unable to perform normal daily activities, missed school or work, or received care (i.e., telehealth, clinic or emergency department visit, or hospitalization) from a medical professional because of new symptoms or conditions.

§§ FDA used the Multi-Item Gamma Poisson Shrinker algorithm to calculate the Empirical Bayes Geometric Mean and its associated 90% confidence interval (EB05, EB95). An EB05 ≥2 (more than twice expected) was considered the threshold for defining a vaccine-event pair reported disproportionately.

¶¶ 45 C.F.R. part 46, 21 C.F.R. part 56; 42 U.S.C. Sect. 241(d); 5 U.S.C. Sect. 552a; 44 U.S.C. Sect. 3501 et seq.

*** Processed VAERS reports are those that have been coded using MedDRA, have been deduplicated, and have undergone standard quality assurance and quality control review.

††† CDC reviewed VAERS reports of syncope for additional information. Syncopal events that occurred off-site or ≥1 hour after vaccine administration were excluded from analysis.

§§§ An updated letter of authorization for the Pfizer-BioNTech COVID-19 vaccine is available at https://www.fda.gov/media/150386/download.
